# Lymphangioléiomyomatose pulmonaire de révélation inhabituelle au cours d'une sclérose en plaque

**DOI:** 10.11604/pamj.2015.20.398.4655

**Published:** 2015-04-23

**Authors:** Neirouz Ghannouchi Jaafoura, Ahmed Guigua, Houneida Zaghouani, Amira Atig, Dajla Bakir, Mabrouk Khalifa, Fethi Bahri

**Affiliations:** 1 Service de Médecine Interne, CHU Farhat Hached, Avenue Ibn, El Jazzar Sousse, Tunisie; 2 Service de Radiologie, CHU Farhat Hached, Avenue Ibn El Jazzar Sousse, Tunisie

**Keywords:** Lymphangioléiomyomatose pulmonaire, embolie pulmonaire, sclérose en plaque, Pulmonary lymphangioleiomyomatosis, pulmonary embolism, multiple sclerosis

## Abstract

La lymphangioléiomyomatose pulmonaire est une pathologie rare de la femme jeune, caractérisée par une prolifération de cellules musculaires lisses immatures, aboutissant à la destruction kystique des poumons avec possibilité d’évolution vers l'insuffisance respiratoire chronique. La découverte est souvent fortuite lors de la prise en charge d'une autre pathologie pulmonaire. Son association à une sclérose en plaque n'a jamais été rapportée, de même que l'embolie pulmonaire in situ comme manifestation inaugurale. Nous rapportons l'observation d'une patiente âgée de 39 ans, suivie pour sclérose en plaque depuis 20 ans, chez qui le diagnostic d'une lymphangioléiomyomatose pulmonaire a été posé, à l'occasion d'une broncho-pneumopathie bilatérale avec une embolie pulmonaire associée motivant la réalisation d'un angioscanner thoracique.

## Introduction

La lymphoangioléiomyomatose (LAM) pulmonaire est une affection rare révélée le plus souvent à l'occasion de manifestations respiratoires peu spécifiques dont les plus évocateurs sont le pneumothorax récidivant, l'hémoptysie et le chylothorax [1]. L'embolie pulmonaire ou les thromboses des artères pulmonaires in situ n'ont jamais été rapportées de même que l'association à une sclérose en plaque (SEP).

## Patient et observation

**Figure 1:**
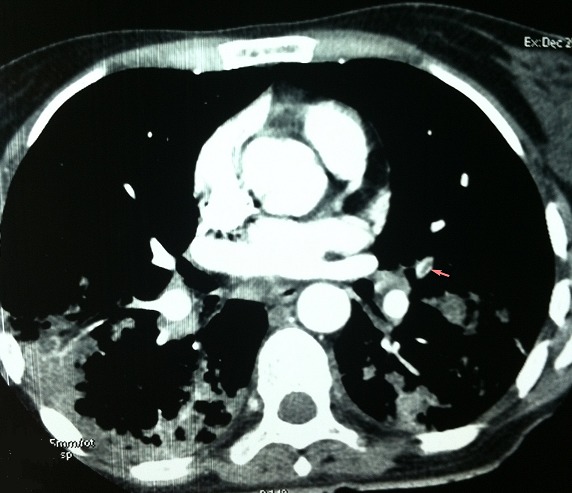
Angio-scanner thoracique - défect endoluminal au niveau de l'artère lingulaire supérieure

**Figure 2:**
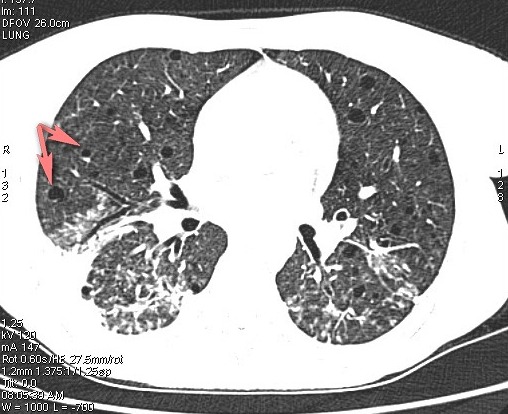
TDM Thoracique - lésions kystiques multiples à paroi fine sur une coupe transversal

**Figure 3:**
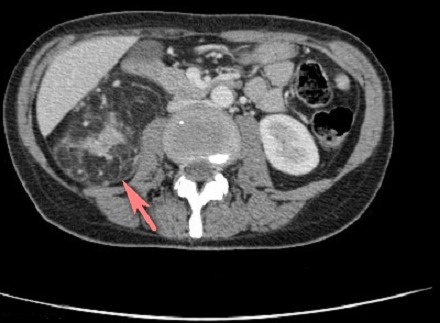
TDM abdominale - masse à point de départ rénal de densité hétérogène avec une composante tissulaire et graisseuse évoquant un angiomyolipome rénal

Mlle B Z âgée de 39 ans, sans antécédents familiaux notables, est suivie depuis l’âge de 19 ans pour une SEP progressive à l'origine d'un handicap moteur. En décembre 2012, elle nous a consulté pour fièvre, toux productive, expectorations purulentes et douleurs basi-thoraciques bilatérales. L'examen initial a objectivé une fièvre à 39°c, un pouls à 120 battements/min, une polypnée à 30 cycles/min, des râles crépitants à l'auscultation pulmonaire et un syndrome tétra-pyramidal, le reste de l'examen physique est normal. La gazométrie à montré une hypoxémie à 56 mmHg et une hypocapnie à 28 mmHg. Le reste du bilan biologique est normal hormis une hyperleucocytose à 14 400 éléments par mm^3^. La radiographie thoracique montre une pneumopathie bilatérale étendue et mal limitée. Dans le doute d'une EP associée, un angioscanner thoracique a été réalisé et a confirmé le diagnostic d'EP en montrant un défect endoluminal lingulaire supérieur et inférieur (Figure 1) ainsi que des lésions kystiques diffuses aux deux poumons évoquant une LAM pulmonaire (Figure 2). L'echo-dopler des membres inférieurs n'a pas objectivé de thrombophlébite. L'IRM cérébro-médullaire, à la recherche de méningiome a montré une atrophie cérébrale et médullaire cadrant avec une SEP avec des hypersignaux de la substance blanche de siège péri-ventriculaire et en sous cortical. Le scanner abdomino-pelvien met en évidence un angiomyolipome rénal gauche de 10 x 8 x 6 cm de grand axe (Figure 3). Le diagnostic d'une LAM dans sa forme sporadique est posé devant les anomalies scanographiques et la présence d'angiomyolipome rénal. Le bilan de thrombophilie, réalisé par ailleurs dans le cadre de l'enquête étiologique de l'EP, est négatif. Une héparinothérapie a été instaurée relayée par un traitement par anti-vitamine K de même qu'une antibiothérapie à large spectre, une réhydratation et des séances de kiné respiratoire avec amélioration de l’état respiratoire et de l’état général.

## Discussion

Nous rapportons la survenue d'une LAM dans l’évolution d'une SEP et dont le diagnostic était posé à l'occasion d'un tableau clinique de broncho-pneumopathie bactérienne compliquée d'embolie pulmonaire sans thrombose veineuse profonde. La LAM est une maladie rare, d'origine encore inconnue, touchant exclusivement les femmes en période d'activité génitale et caractérisée par une prolifération de cellules musculaires lisses immatures au niveau des espaces péri bronchiolaires, péri lymphatiques et/ou péri vasculaires entraînant une destruction kystique progressive des poumons, avec un risque d’évolution vers une insuffisance respiratoire chronique sévère en 10 à 15 ans [1]. Cette maladie se voit dans le cadre d'une sclérose tubéreuse de Bourneville où environ 40% des femmes ont une LAM et peut être sporadique avec une prévalence de 2 à 5 cas /million d'habitants. La survenue au cours d'une SEP n'a jamais été rapportée dans la littérature. Divers symptômes respiratoires peuvent révéler la maladie. Il peut s'agir d'une dyspnée d'effort observée dans 70% des cas, d'une toux ou d'une douleur thoracique [2]. Elle peut être diagnostiquée à l'occasion d'une complication et en particulier de pneumothorax, révélateur dans 40% des cas et pouvant être récidivant, d'hémoptysie dans près de 15% des cas et parfois de chylothorax dans 10% des cas. Des complications thrombo-emboliques ont rarement été rapportées [3, 4], mais une thrombose in situ, tel est le cas de cette patiente, n'a jamais été décrite. D'un autre côté, la SEP ne constitue pas une situation à risque d’évènements thrombo-emboliques [5]. Des manifestations extra-pulmonaires de la LAM peuvent se voir et sont dominées par les angiomyolipomes rénaux présents chez 50% environ des patientes atteintes de formes sporadiques [1, 2]. Le diagnostic de certitude repose sur l'association d'une présentation radio-clinique évocatrice et d'une confirmation histologique obtenue par biopsie pulmonaire, celle-ci ne peut être envisagée chez notre patiente vue qu'elle est traitée par anti-coagulants pour l'EP. Lorsqu'une biopsie ne peut être pratiquée, des critères pour le diagnostic de la LAM ont été établis et le critère histologique peut être remplacé par la présence d'angiomyolipome du rein, d’épanchement chyleux thoracique ou abdominal ou de la présence de lymphangioléiomyome ou d'adénopathies atteintes par la LAM [6].

## Conclusion

Cette observation est originale par deux particularités, l'association inédite d'une LAM à une SEP et le mode de révélation inhabituel qui est la thrombose in situ des artères pulmonaires. Grace au développement des techniques de radiologie, un diagnostic précoce de cette affection est devenu possible, en l'absence de manifestations cliniques spécifiques.
